# Worker-dependent gut symbiosis in an ant

**DOI:** 10.1038/s43705-021-00061-9

**Published:** 2021-10-28

**Authors:** Hiroyuki Shimoji, Hideomi Itoh, Yu Matsuura, Rio Yamashita, Tomoyuki Hori, Masaru K. Hojo, Yoshitomo Kikuchi

**Affiliations:** 1 Shool of Biological and Environmental Sciences, Kwansei Gakuin University, Sanda, Hyogo Hyogo 669-1337 Japan; 2 Bioproduction Research Institute, National Institute of Advanced Industrial Science and Technology (AIST) Hokkaido, Sapporo, Hokkaido 062-8517 Japan; 3 Tropical Biosphere Research Center, University of the Ryukyus, Nishihara, Okinawa 903-0213 Japan; 4 Environmental Management Research Institute, National Institute of Advanced IndustrialScience and Technology (AIST) Tsukuba West, Tsukuba, Ibaraki 305-8569 Japan

**Keywords:** Ecology, Evolution

## Abstract

The hallmark of eusocial insects, honeybees, ants, and termites, is division of labor between reproductive and non-reproductive worker castes. In addition, environmental adaption and ecological dominance are also underpinned by symbiotic associations with beneficial microorganisms. Microbial symbionts are generally considered to be maintained in an insect colony in two alternative ways: shared among all colony members or inherited only by a specific caste. Especially in ants, the reproductive caste plays a crucial role in transmission of the symbionts shared among colony members over generations. Here, we report an exceptional case, the worker-dependent microbiota in an ant, *Diacamma* cf. *indicum* from Japan. By collecting almost all the individuals from 22 colonies in the field, we revealed that microbiota of workers is characterized by a single dominant bacterium localized at the hindgut. The bacterium belonging to an unclassified member within the phylum Firmicutes, which is scarce or mostly absent in the reproductive castes. Furthermore, we show that the gut symbiont is acquired at the adult stage. Collectively, our findings strongly suggest that the specific symbiont is maintained by only workers, demonstrating a novel pattern of ant-associated bacterial symbiosis, and thus further our understanding of host-microbe interactions in the light of sociobiology.

## Introduction

Symbiotic bacteria affect the fitness of the host animals by altering the life cycle, reproductive system, nutritional status, and physiological/behavioral traits in a wide range of taxa [1–3]. Among them, extensive studies have shown remarkable diversity of symbiotic relationships between insects and bacteria, in which novel biological functions, transmission modes, and co-evolutionary processes of the symbioses have been unraveled [4]. Obligate symbiosis, such as the aphid-*Buchnera* symbiosis, is one of the spectacular events of the major evolutionary transitions from eukaryotic cells to more complex organisms [5]. Ecological and evolutionary consequences of symbioses should extend not only to the host individual but also to the group of animals such as colonies of eusocial insects [6].

The ecological success of ants has been achieved by the evolution of sophisticated eusociality, but also underpinned by symbiotic relationships with other associated organisms [7–9]. Many ants are known to protect sap-feeding hemipteran insects from predators in exchange for honey dew as a stable nutritional resource. Meanwhile, ant-bacterial symbiosis is one of the most prominent systems to study the intricate beneficial co-evolution of host-microbe partnerships. Carpenter ants in the genus *Camponotus* species harbor obligate *Blochmannia* symbionts in bacteriocytes [10]. The *Blochmannia* symbionts produce essential amino acids and few co-factors for ants, which are tightly involved with the survival of a colony [11]. In the leaf-cutter ants, known for farming fungi as food, ectosymbiotic bacteria (genus *Pseudonocardia*) localized on the exoskeleton of colony members release antibiotics and prevent specialized garden-parasite (genus *Escovopsis*) from eroding the fungal garden [12]. Moreover, as an example of phenotypic change caused by symbiotic bacteria, Russell et al. [ref. 13] suggest that gut symbionts have mediated the evolution of herbivory in ants by supplying nutrients. Thus, bacterial symbiosis underlies ecological success of ant societies in various manners.

To understand the evolution of such long-lasting mutualistic relationships, identifying the transmission mode of symbiotic bacteria is essential [14]. There are two types of transmission patterns of symbiotic bacteria: inter-generational transmission (i.e., vertical transmission from mother to offspring) and intra-generational transmission (i.e., horizontal transmission between individuals). In case of eusocial Hymenoptera, especially social bees and ants establish complex societies based on the sophisticated division of labor between reproductive and non-reproductive worker castes [15]. Hence, the queen harboring beneficial bacteria constructs a new nest (inter-generational transmission) [10, 12], and then the bacteria are transferred to other colony members (intra-generational transmission) [16, 17]. As a result, the symbiotic bacteria are maintained within the colony, whose pattern is further divided into two conceivable ways. One is that all colony members share beneficial bacteria regardless of castes [10, 12, 18–20]. The other is that the microbiota is specialized depending on the castes as shown in a fungus-farming termite *Macrotermes natalensis* [21]. In most cases, but see honeybee [20], the symbionts of reproductive castes are transferred to the next generation, confirming that the reproductive individual is considered to play a crucial role in the symbiont transmission process [10, 12, 21]. Nevertheless, recent studies using high-throughput DNA sequencing technology have focused on the diversity of microbial community only in workers of various ant species [22]. In spite of the significance of symbiont transmission mode to the next generation for understanding co-evolutionary patterns, most of these studies have neglected the symbionts of those reproductive caste in ant societies due to the sampling bias in an open field.

*Diacamm*a cf. *indicum* from Japan belonging to the primitively eusocial Ponerinae ants [23, 24] is the only species of genus *Diacamma* in the Japanese archipelago, more specifically Okinawa Island. A colony consists of one gamergate, a mated egg-laying worker (functionally equivalent to queen) [25], and 50-300 workers [26]. In this species, the reproductive division of labor is regulated by surgical manipulation; all individuals can differentiate into any caste at adult stage; however, the gamergate bites off thoracic pared-appendages (gemmae) of newly eclosed workers using its mandibles; the resultant individuals cannot mate and differentiate into the worker caste irreversibly [27]. A colony replicates by fission (the division of colony members into two or more sub-groups), resulting in two types of colonies, in which either the colony has the gamergate or not. Subsequently, a new sub-colony with brood items (eggs or larvae) is destined to lack the gamergate. In that gamergate-absent colony, a newly emerged worker possessing gemmae mates with a male and monopolizes reproduction as the gamergate. In this species, behavioral caste is largely determined by age [28, 29]: younger workers are engaged in raring blood items (eggs or bloods) as “nurses” inside a nest, and older workers are involved in foraging outside the nest as “foragers.” Accordingly, the ages of workers can be estimated by the typical roles [30]. As a model species of insect societies, *D*. cf. *indicum* has been often used to manipulate the colony composition and reproduction, providing us a tractable system to carry out several types of behavioral observations and physiological experiments [30–34].

In this study, we examine the intra-colonial diversity of the microbiota in *D*. cf. *indicum*. Our results highlight the presence of a worker specific bacterium, and we discuss how the symbiont is maintained in the ant colony over generations.

## Materials and methods

### Field collection of ant species

**Fig. 1:**
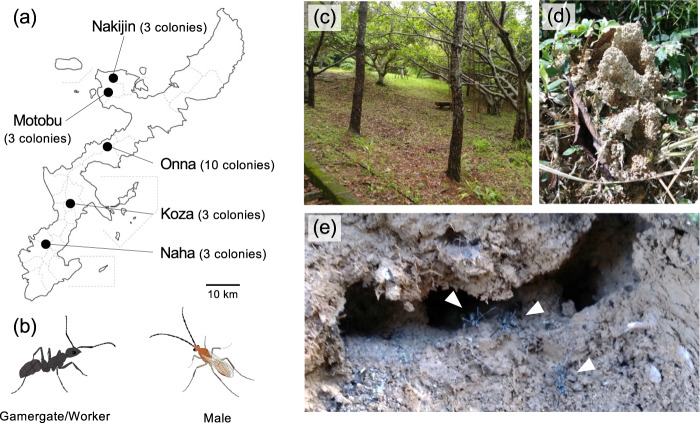
Habitat and morphology of *Diacamma* cf. *indicum* from Japan. **a** Geographical locations for designating populations of *Diacamma* cf. *indicum* sampled in this study. Numbers in parentheses indicate the number of colonies used in the deep sequencing analysis (Table S1). *Diacamma* samples at larval and adult stages were collected in Naha and Onna (Table S2). **b** Illustrations of general morphologies of a gamergate/worker (distinguished by the presence/absence of thoracic appendages) and a male. **c** A sparse forest or grassy slope where ants build their nests. **d** Entrance of the ant nest. **e** Ants (triangles) coming out of a destroyed nest.

We collected 2–5 foragers from each 22 colonies in five populations (Naha, Koza, Onna, Motobu, and Nakijin) in Okinawa main Island (Fig. 1a; Table S1), and stored them directly into vials filled with acetone. Then, we captured all remaining colony members alive and brought them to the laboratory. We checked gemmae of each individual (see *Introduction*) under the dissecting microscope for identification of gamergates, after which a gamergate from each colony was stored separately in a plastic tube filled with acetone. In case males were collected, they were also sampled in the same manner as above. In total, we collected 105 workers, 22 gamergates and 8 males for a deep sequencing analysis of microbiota as described below (Fig. 1b). Also, we sampled 19 larvae, 5 pre-pupa, 17 pupa, and 30 nurses (the younger workers) of six colonies from two populations for quantification of resident bacteria (Table S2).

### Deep sequencing of 16S rRNA gene

After whole body of each insect sample was homogenized with a pestle, DNA was extracted from the resulting homogenates using QIAmp DNA mini kit (Qiagen, Hilden, Germany) according to the manufacture’s instruction for “DNA Purification from Tissues” with an additional digestive step before the proteinase treatment. Using the prepared DNA, the variable region V4 of bacterial 16S rRNA gene was amplified by PCR. Paired-end sequencing for all PCR amplicons was performed on Illumina MiSeq sequencer (Illumina) with MiSeq Reagent kit v2 (Illumina) according to the manufacture’s instruction. Raw sequences data was preprocessed as performed as described previously [35, 36]. The resulting sequences were subjected to taxonomic assignment and clustering based on operational taxonomic unit (OTU) with 3% differences by using RDP classifier ver. 2.11 with a 50% confidence threshold and the macqiime ver. 1.9.1, respectively [37, 38] (see Supplementary Information).

### Sanger sequencing of 16S rRNA gene

For three ants in one colony derived from five populations, using extracted DNA from whole body of each insect as described above, bacterial 16S rRNA gene was directly amplified by PCR using universal primers for bacterial 16S rRNA gene, 27F and 1492R (Table S3), and AmpliTaq Gold 360 master mix (Applied Biosystems, CA, USA) according to the manufacture’s instruction, with the following thermal cycling conditions: initial denaturation at 95 °C for 10 min, followed by 30 cycles of 95 °C for 30 s, 55 °C for 60 s, and 72 °C for 90 s, and a final extension at 72 °C for 120 s. All the PCR products (in total 15) were purified and sequenced using a Sanger sequencer as described previously [39] (See Supplementary Information). The resulting fragmented sequences were assembled using ATSQ tool version 5.4.1 in genetyx-mac version 19 (Software Development, Tokyo, Japan). In addition, in order to validate the sequences obtained from direct PCR amplification above and prepare a plasmid template used for quantitative PCR analyses below, PCR amplicon derived from a randomly selected forager, which was collected in Onna, was sub-cloned as described previously [40]. A PCR product was prepared using universal primers for bacterial 16S rRNA gene, 27F and 1492R, with extracted DNA from a forager as described above, and cloned with competent cells of *Escherichia coli* DH5α (Takara, Otsu, Japan) and pT7Blue T-vector (Novagen, WI, USA). The plasmid insertion was sequenced with the primers Univ19, 926R, and Rev20 (Table S3) on a Sanger sequencer and assembled as described above.

### Quantitative PCR

Quantitative PCR (qPCR) was performed to amplify partial 16S rRNA gene sequence of unclassified Firmicutes using specific primers, DiaUFF and DiaUFR (Table S3 and see also *Primer and probe design for qPCR and wFISH* in Supplementary Information), and StepOnePlus (Applied Biosystems, CA, USA) with THUNDERBIRD SYBR qPCR mix (Toyobo, Osaka, Japan) according to the manufacture’s instruction. A standard curve was created using a ten-fold dilution series from 10^1^ to 10^8^ copies/µl of the plasmid solution inserted with almost-complete 16S rRNA gene sequence from Firmicute symbiont (DDBJ Accession no. LC625538) prepared from sub-cloning analysis as described above. The thermal cycling for all genes were performed as follows: preheat at 95 °C for 30 s; 40 cycles of 95 °C for 5 s, 58 °C for 30 s, and 72 °C for 30 s.

### Phylogenetic analysis

The multiple alignment of nucleotide sequences of 16S rRNA gene was constructed by the program MAFFT v7.475 [41] with --globalpair and --maxiterate 100 options, and reliability of the aligned nucleotide sites was evaluated by the program GUIDANCE2 [42], in which the sites with confidence scores at 0.804 or lower were removed from the alignment. After manually checking and removing the gaps, in total, 1,262 sites were used in the following analyses. We performed two phylogenetic analyses, Bayesian (BA) with MrBayes v3.2.7 [43] and maximum likelihood (ML) with RAxML-NG [44], and each analysis was conducted under the nucleotide substitution model GTR + I + G4 determined by the program Modeltest-NG [45] based on the AIC, AICc and BIC criteria. In the BA analysis, two independent runs with 20 simultaneous Markov chains were performed for 10,000,000 generations producing 7500 trees in total (sample freq = 1000, burnin = 2501, mcmc temp = 0.2 for each run). The trees were used to generate a majority consensus tree and calculate the posterior probabilities. In the ML analysis, the best-scoring ML tree was searched from 50 distinct parsimony starting trees, and then 1,000 bootstrapping were conducted for generating support values. The ML support values were then mapped on the tree generated by BA for a comparison. The resulting tree, statistical values and OTU information were illustrated and edited in FigTree v1.4.4 (https://github.com/rambaut/figtree/releases/tag/v1.4.4) and Adobe Illustrator 2021 (Adobe Inc.).

### Whole-mount fluorescence in situ hybridization (wFISH)

wFISH was performed in order to detect the localization of the unclassified Firmicutes in the abdomen of *D*. cf. *indicum* by following the protocol described previously [46] with minor modifications in the addition of helper oligonucleotides, temperature of hybridization and an extra washing step before counterstaining. The whole gut tissues including hindgut and rectum of fresh adult *D*. cf. *indicum* (Fig. 1a) of more than 25 samples collected in Onna, were dissected in PBS (137 mM NaCl, 2.7 mM KCl, 8.1 mM Na_2_HPO_4_, 1.5 mM KH_2_PO_4_ [pH 7.4]) under the dissecting microscope Leica S8APO (Leica Microsystems), washed in 70% ethanol twice, and fixed in Carnoy’s solution (ethanol-chloroform-acetic acid [6:3:1]) overnight. After rinsing the tissues with absolute ethanol three times, they were bleached by 6% hydrogen peroxide in 80% ethanol at 4 °C for 2 weeks to 1 month to quench the autofluorescence [47] by replacing the solution every week. Before hybridization, the tissues were thoroughly washed in 70% ethanol once and PBSTw (PBS containing 0.05% Tween20) three times. The samples were then pre-hybridized in hybridization buffer (20 mM Tris-HCl [pH 8.0], 0.9 M NaCl, 0.01% sodium dodecyl sulfate, 30% formamide). After replacing the solution twice, the samples were incubated with the hybridization buffer containing 100 nM of AlexaFluor fluorochrome-labeled oligonucleotide probes (Molecular Probes® ThermoFisher Scientific) and helper oligonucleotides targeting the unclassified Firmicutes (listed in Table S3 and see also *Primer and probe design for qPCR and wFISH* in Supplementary Information) at 37 °C overnight. The excessive probes bound to non-specific sequences were washed off by incubation in fresh hybridization buffer at 42 °C for 30 min, and then the tissues were counterstained by DAPI [4,6-Diamidino-2-phenylindole, dihydrochloride] (DOJINDO Laboratories) at a concentration of 1 ng/ml and/or SYTOX^®^ Green (Thermo Fisher Scientific) at a concentration of 1:10000 in PBSTw for 15 min. After washing with PBSTw twice, the tissues were mounted in Slowfade antifade solution (Invitrogen^®^ Thermo Fisher Scientific) and observed with a confocal laser scanning microscope C2si (Nikon).

### Availability of nucleotide sequence data

Sequence data of the bacterial 16S rRNA genes derived from insect microbiota have been deposited in the DDBJ/Genbank/EBI databases under accession numbers PRJNA722170 for the deep sequencing data set and LC623637-LC623651, LC625538, and LC625539 for Sanger sequencing data.

## Results

### Amplicon and sanger sequencing of *D*. cf. *indicum* microbiota reveal a Firmicute-dominant community

**Fig. 2:**
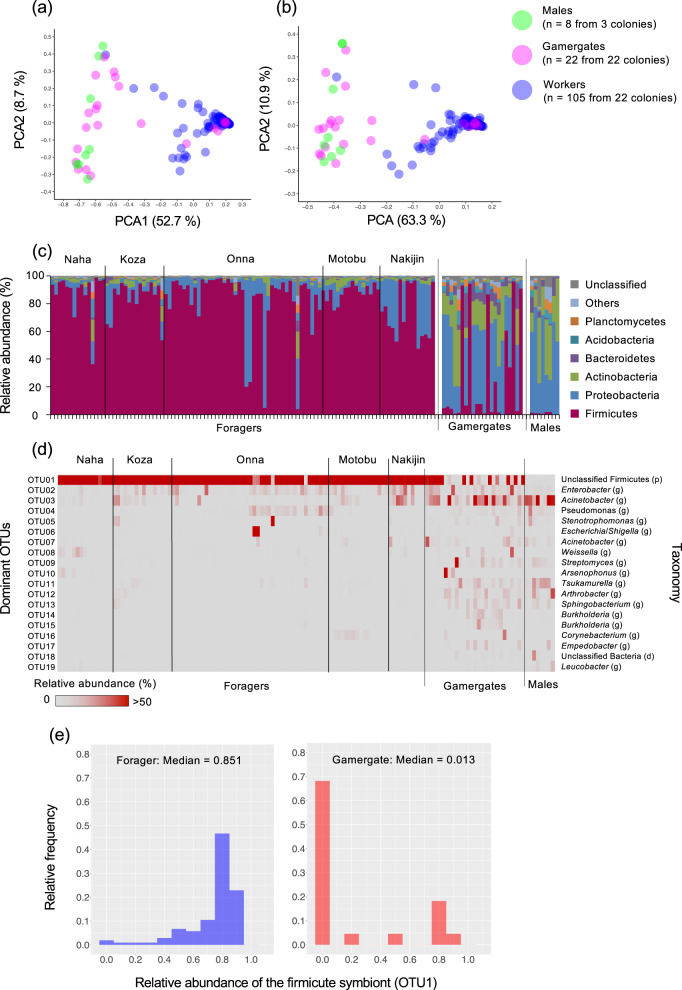
Diversity and specificity of ant-associated microbiota in each caste, forager (*n* = 105), gamergate (*n* = 22), and male (*n* = 8), based on a deep sequencing analysis of partial 16S rRNA sequences. Dissimilarity of community structure of microbiota in each caste shown by Bray-Curtis (**a**) or the weighted UniFrac (**b**) Principal Coordinate Analysis (PCoA) plot based on the same amount of sequences (10,000). **c** Community structure of each microbiota expressed by the relative abundance at the rank of phylum. **d** Relative abundance and taxonomy of dominant OTUs with more than 1.0% of relative abundance in any of the three castes. **e** Histograms showing relative abundance of unclassified Firmicutes (OTU01) among foragers (left panel) and gamergates (right panel).

To reveal the diversity of microbiota associated with *D*. cf*. indicum* from Japan, we examined and compared community structure of microbiota for foragers, gamergates, and males. PCoA plots based on Bray-Curtis and the weighted UniFrac distance showed that community structures of forager’s microbiota significantly differed from those of gamergate’s and male’s (PERMANOVA: Bray-Curtis distance: foragers vs gamergate: *F* = 50.556, *r*^2^ = 0.288, *P* < 0.0001; foragers vs males: *F* = 70.146, *r*^2^ = 0.387, *P* < 0.0001; unifrac distance: foragers vs gamergates: *F* = 66.422, *r*^2^ = 0.346, *P* < 0.0001; foragers vs males: *F* = 84.774, *r*^2^ = 0.433, *P* < 0.0001; Fig. 2a, b). On the other hand, there was no statistically significant difference of the community structure between gamergate and male (Bray-Curtis distance: gamergates vs males: *F* = 2.286, *r*^2^ = 0.075, *P* = 0.029; unifrac distance: gamergates vs males: *F* = 2.112, *r*^2^ = 0.070, *P* = 0.069; Fig. 2a, b). Note that we used an adjusted *P* value (*α* = 0.05/3 = 0.017) by Bonferroni correction for post-hoc comparisons (see Supplementary Information). At the bacterial phylum level, dominance of Firmicutes (≥50% relative abundance) were observed in 99/105 foragers samples (94%), 6/22 gamergate samples (27%), and 0/8 male samples (0%) (Fig. 2c). Proteobacteria and Actinobacteria were major groups in microbiota of foragers (6/105) and gamergates (16/22) with less Firmicutes (<50% relative abundance), and the same tendency was observed in those of all males (Fig. 2c). OTU-based analysis showed that microbiota of 99/105 foragers samples (94%), 6/22 gamergate samples (27%) were occupied by one extreme dominant OTU (OTU01; ≥ 50% relative abundance; Fig. 2d, e), belonging to unclassified member of the phylum Firmicutes (hereafter, firmicute symbiont). In stark contrast to most foragers (99/105, 94%), microbiota of 16/22 gamergates and all males consisted of Proteobacteria (the genera *Enterobacter*, *Acinetobacter*, *Pseudomonas*, *Arsenophonus*, etc) and Actinobacteria (the genera *Streptomyces*, *Tsukamurella*, *Arthrobacter*, etc) OTUs, instead of the firmicute symbiont (OTU01) (Fig. 2c, d, Table S4). Next, we obtained almost full length 1.5-kb 16S rRNA gene of the firmicute symbiont by directly Sanger sequencing the amplified products of each three foragers from five studied sites (Table S1). All obtained 15 sequences, 1,443 bp in length, were identical to each other and contained an identical sequence derived from the most redundant OTU01 above and from a forager in Onna for sub-cloning analysis. Such reproducible sequence showed the highest BLASTn hit to a bacterial 16S rRNA gene of Firmicutes bacterium isolate CS421 from the ant *Leptogenys* sp. [KX983336] (94.95% [1317/1387]). Based on this sequence of the firmicute symbiont, we designed specific primers and probes in the following qPCR and wFISH analyses. Another type of clone K5O3 obtained from an individual collected in Onna, 1484 bp in length, showed the highest BLASTn hit to an uncultured bacterium clone ncd2518b07c1 [JF216746] (98.84% [1278/1293]) derived from the human skin, but was detected in only one forager among all 135 individuals used in deep sequencing analysis and not identical to sequences of any OTUs shown in Fig. 2d.

### Density of the firmicute symbionts in different castes of *D*. cf. *indicum*

**Fig. 3:**
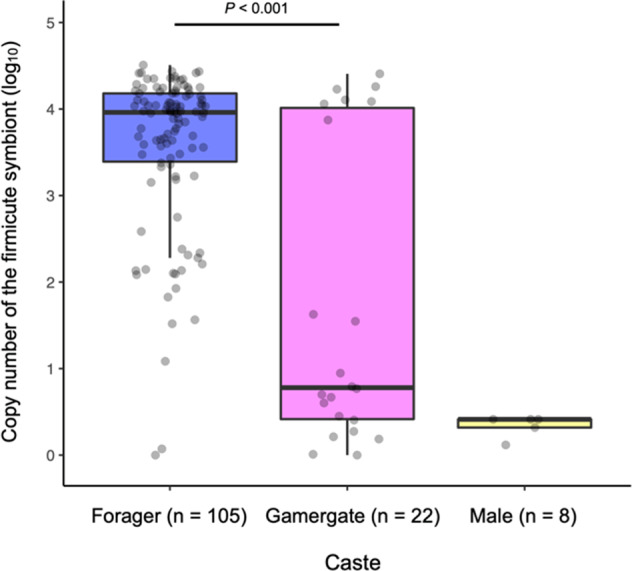
Absolute abundance of the firmicute symbiont in each caste, estimated by qPCR analysis. Males were excluded from statistical analysis because of almost no detection.

Deep sequencing analysis revealed that foragers harbored much more skewed microbiota than the other castes; the exclusive dominance of firmicute symbiont. Here, the absolute abundance of firmicute symbiont was estimated by qPCR analysis with a specific primer set. Copy numbers of 16S rRNA gene for the firmicute symbiont in foragers was for a median of 9.147 × 10^3^ copies/ individual, which was significantly higher than that in gamergates (median = 5 × 10^0^ copies/ individual) (Linear mixed model (LMM); *χ*^2^ = 74.37, *P* < 0.0001, Fig. 3). In case of males, that values of the firmicute symbiont were extremely low (<10^1^ copies/individual, Fig. 3). Note that statistical values for the constructed LMM, estimated coefficients with 95% CI, variances of random effect, estimated dispersion parameter, effect size, and AIC, are shown in Table S5.

### Phylogenetic placement of the firmicute symbiont

**Fig. 4:**
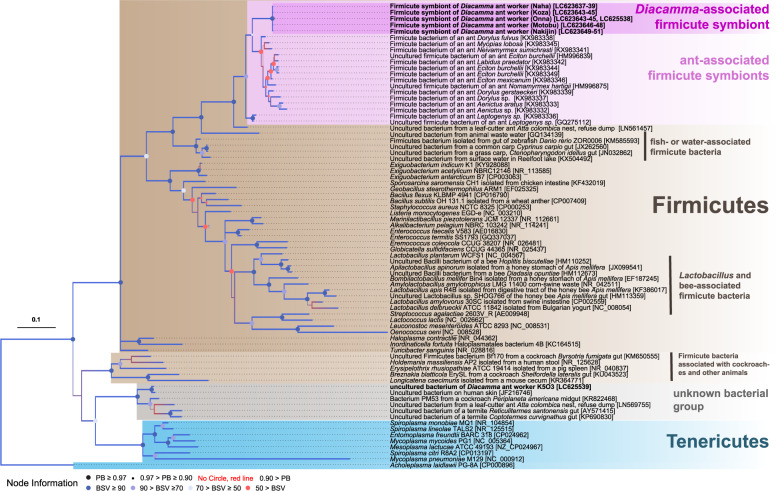
Phylogenetic placement of firmicute symbiont of *D*. cf. *indicum* based on the nucleotide sequences of 16S rRNA gene. The other bacterial sequences belonging to Firmicutes, Tenericutes and the unknown bacterial group were retrieved from GenBank, trimmed, and aligned, producing in total 1,262 sites. A Bayesian phylogeny is displayed with posterior probabilities (PB) depicted by the size of circles or their absence at the nodes, while bootstrap support values (BSV) were represented by colors of the circles. See the bottom of the figure for those symbols reflecting specific numeric values. OTUs in boldface type indicate sequences obtained in this study with collection sites in parentheses and accession numbers in brackets. Sequences of worker K5O3 were obtained from the clone library analysis, and the others from the direct sequencing analysis of 3 workers for each colony. The scale bar represents the branch length corresponding to the number of substitutions per site.

In order to infer the phylogenetic placement of the firmicute symbiont from *D*. cf. *indicum* in Okinawa, we conducted two kinds of molecular phylogenetic analyses based on 16S rRNA gene sequences from five different sites (Fig. 4). Our results showed that the bacterial symbiont of *D*. cf. *indicum* was consistently placed within the phylum Firmicutes and tightly clustered with Firmicutes bacteria derived from various field-collected ant species in the subfamilies Dorylinae and Ponerinae (BA posterior probability: 1.00; ML bootstrap support value: 77) [13, 48, 49]. The closest relative of this bacterial group was detected from the leaf-cutter ant *Atta colombica* nest refuse [50]. Then, this whole group of ant-associated symbiotic bacteria was allied to several uncharacterized bacteria associated with fish gut or detected from environmental water, indicating that the evolutionary origin of this ant-Firmicutes association is independent from other insect-associated Firmicutes bacteria such as *Lactobacillus*, *Apilactobacillus* and *Bombilactobacillus* spp. in bees and *Breznakia blatticola* and an unnamed bacterium in cockroaches.

In the same analyses, another unique sequence determined from the clone K5O3 of *D*. cf. *indicum* in Onna was placed within an unknown bacterial group between two phyla, namely Firmicutes and Tenericutes (Fig. 4 below, in gray). The closest relative of the clone was uncultured bacterium on human skin, but the other allied species contain another bacterium from the leaf-cutter ant refuse, several gut bacteria from a cockroach *Periplaneta americana* and few termites [50–52]. Due to extremely low occurrence and paucity of phylogenetic data of this bacterial group, however, we abandoned further analyses on the host-specificity or possible DNA contamination of this bacterium in this study.

### In situ detection of the firmicute symbiont in *D*. cf. *indicum* workers

**Fig. 5:**
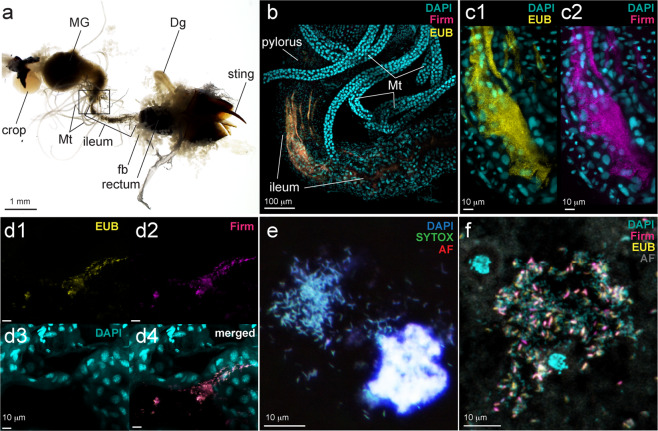
Visualization of *in vivo* localization of the firmicute symbiont in foragers of *D*. cf. *indicum*. **a** A light microscopic image of dissected abdomen of a forager. **b** A merged confocal image detecting wFISH signals of a dense bacterial population in the ileum adjacent to Malpighian tubules, in which magenta represents the firmicute symbiont specific probe (Firm) and yellow indicates universal eubacteria probe (EUB). Host nuclei were stained by DAPI and shown as cyan. **c** (1) A close-up image of (**b**) showing only EUB signals and (2) the same focal plane showing Firm signals. **d** A small population of bacteria at the posterior part of the ileum close to rectum representing (1) EUB, (2) Firm, (3) DAPI, and (4) merged, respectively. **e** Smeared bacterial cells from the dissected ileum of a forager stained by DAPI and SYTOX Green, alongside an unknown substance also detected by its auto-fluorescence (AF, red). **f** A merged FISH image of the hindgut content smeared on a glass slide. The unknown substance was visualized by detecting its auto-fluorescence using the laser diode 488 nm and pseudocolored in gray (AF). Dg Dufour’s gland, fb fat body tissues, HG hindgut, MG midgut, Mt Malpighian tubules.

We dissected *D*. cf. *indicum* workers and isolated most of the gastrointestinal tracts along with fat body tissues, Dufour’s gland, venom reservoir and sting attached (Fig. 5a), which were then fixed, bleached and subjected to fluorescence in situ hybridization of the firmicute symbiont and eubacteria. We especially focused on the gut compartments of foragers which contained some dark food particles presumably being digested in the gut. wFISH of foragers clearly showed densely populated bacterial mass as co-localized signals of both firmicute-specific and universal probes especially at the anterior end of the ileum (Figs. 5b and c1-2). On the other hand, crop, midgut, Dufour’s gland, rectum and venom reservoir did not contain any visible signals of the firmicute symbiont (Fig. S1a-i) although there were few individuals with some clusters of bacterial signals by the universal probe only in the midgut (Fig. S1d). When we observed closely at more posterior parts of ileum, there were several patchy populations of the firmicute symbiont detected by the same probes (Fig. 5d1-4 and Fig. S1h). The morphologies of these cells were partially captured but still lacking clarity; therefore, we specifically isolated the gut contents from the ileum onto glass slides and observed bacterial cells via DNA staining and FISH (Fig. 5f and g). Small filamentous bacterial cells of about 1–2 μm in length were successfully visualized with the probes, and the cells were often tightly aggregated and attached to some unknown substance emitting strong auto-fluorescence (Fig. 5f).

### Prevalence of the firmicute symbiont in each developmental stage of *D*. cf. *indicum*

**Fig. 6:**
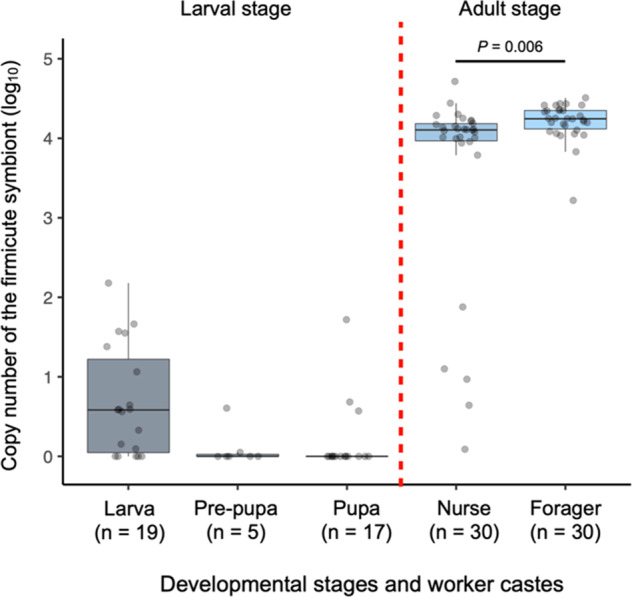
Absolute abundance of the firmicute symbiont in the developmental stages and worker castes of *D*. cf. *indicum*, estimated by qPCR analysis. Larva, pre-pupa, and pupa were excluded from the statistical analysis because no detection was observed in most samples.

Prevalence of the firmicute symbiont in each developmental stage of *D*. cf. *indicum* were examined by the same qPCR analysis used for the individuals in the deep sequencing analysis. Copy numbers of 16S rRNA gene of the firmicute symbiont in almost all specimens at larval stage (i.e., larva, pre-pupa, and pupa) were <10^2^ copies/individual or undetectable, which were extremely smaller than those in nurses and foragers (Fig. 6). At adult stage, that values of most nurses and all foragers were around 10^4^ copies/individual, however, those of several nurses (5/30 (applicable/total)) were <10^2^ copies/individual, which was the same level to those of larval stage specimens (Fig. 6). Precisely, our statistical analysis showed that the foragers harbored higher titer of the firmicute symbiont than the nurses (LMM; *χ*^2^ = 7.679, *P* = 0.006, Fig. 6). Note that all statistical values for the constructed LMM are shown in Table S6.

## Discussion

In this study, by focusing on a caste system of eusocial Hymenoptera, we investigated diversity and specificity of microbiota in *Diacamm*a cf. *indicum* from Japan. Our results unequivocally revealed that community structure of forager’s microbiota was occupied by single OTU belonging to unclassified members in Firmicutes, although the relative and absolute abundance of this OTU were scarce or absent in many gamergates (Figs. 2 and 3). Phylogenetic analyses of the firmicute symbiont from five different populations revealed that *Diacamma* symbionts form a distinct clade and belong to an unnamed bacterial taxon consisting of symbionts associated with predatory ant species, such as army ants (Fig. 4). Our microscopic observation of the firmicute symbiont in the gastrointestinal tracts showed that the symbiont is densely localized at the ileum of the hindgut in foragers (Fig. 5). Finally, our quantitative analysis revealed that the firmicute symbiont was almost absent in pre-adult stages (Fig. 6). Collectively, these results demonstrate that the firmicute symbiont identified in this study is a gut-associated symbiont of *D*. cf. *indicum*, and markedly dominant in workers.

As shown in the phylogenetic analyses (Fig. 4), the firmicute symbiont has been identified in New World army ants and is highly prevalent in *Eciton* and *Labidus* species [49]. Although the previous work revealed that the firmicute symbiont is one of the core gut microorganisms of the predatory ants, only workers were inspected. Our study not only corroborated the fact that the firmicute symbiont is predominantly maintained in the worker caste but also demonstrated that it is not in the reproductive castes, exemplifying a clear-cut case of caste-dependent symbiotic association. Recently, a caste-dependent microbiota has been reported in the Japanese carpenter ant *Camponotus japonicus* [53], where highly diverse microbiota was only detected in male individuals. Therefore, to our knowledge, this is the first evidence of the worker-dependent microbiota/symbiont in ants.

Group-living animals can obtain various benefits from not only group foraging or defense, but also sharing beneficial bacteria [54]. In eusocial insect societies, the symbionts are horizontally transmitted to colony members by social interactions [55, 56]. As a result, shared symbiotic bacterial communities that entail novel biological functions bring benefits to colonies as a whole [12, 16, 17, 20, 57, 58]. In ants, such beneficial partners are transferred to the next generation through reproductive castes [10, 12]. For example, intracellular symbiont *Blochmannia* of Camponotini is vertically transmitted to offspring from the queen via ovarial infection route, which are known to have considerably altered developmental gene expression patterns in host embryos [59–61]. In leaf-cutter ants, alate queens carry the antibiotics-producing *Pseudonocardia* symbiont and transfer it to colony members in newly founded nests [12]. In stark contrast to previous reports, we showed that *D*. cf. *indicum* gamergates (functional queens) and males scarcely harbored the firmicute symbiont but the infection is confined to workers. These results strongly suggest that reproductive castes would not be involved in the maintenance and transmission processes of the gut symbiont while workers would play a crucial role for maintaining and transferring the firmicute symbiont.

Notably, the specific swarming (colony fission) behavior of *D*. cf. *indicum*, we suspect, could allow this worker-dependent transmission of the firmicute symbiont. A colony of *D*. cf. *indicum* replicates by fission of colony members accompanying eggs and pupae, and a newly emerged individual becomes a gamergate in a daughter colony [27]. Subsequently, old workers within the sub-colony could transfer the firmicute symbiont to new workers produced by the new gamergate through social interactions, such as contact to feces [e.g., ref. 16]. Therefore, in this proposed system, the symbiont could be transmitted to the next generation, as a kind of “pseudo-vertically” transmission [62], without any direct transmission via reproductive caste. Interestingly, in *Apis mellifera*, where colonies are known to replicate by fission, it is considered that workers transmit symbiotic bacteria to the next generation [20]. Of course, an alternative route of the symbiont transmission could be taken into account: the firmicute symbiont might not be transmitted pseudo-vertically among colony members but acquired from the surrounding environment by the foragers. Such horizontal transmission is well known in solitary insect species, such as the bean bug [40, 63]. The environmental transmission hypothesis could also explain why the symbiont titer was high in foragers (workers outside the nest) and was slightly lower in nurses (workers inside the nest) (Fig. 6). In addition to above infection routes only considering the host side, specific traits of the symbiont may also facilitate the infection of workers. Interestingly, a comparison between nurses and foragers showed that five out of 30 nurses have lower-level of the firmicute symbiont, which suggest that the firmicute symbiont proliferates rapidly to a certain level in the gut after being acquired by the young workers (Fig. 6). Therefore, we cannot exclude a possibility that the firmicute symbiont has some remarkable mechanisms to specifically infect ants as seen in entomopathogenic fungi [64–66], which might facilitate the acquisition of the symbiont by either social interaction or environmental transmission. Further study should examine the above hypotheses to uncover the nature of the acquisition mechanism in workers of *D*. cf. *indicum*.

Our results indicated that some gamergates harbor high titer of the firmicute symbiont (Figs. 2, 3), which suggests that the symbiont can be transferred to even the gamergate. However, why is the firmicute symbiont not transmitted to and maintained in most gamergates? There are several possible mechanisms to maintain the asymmetric relationship between castes based on its social life. Generally, reproductive castes, brood items, and nurses are located deep inside the nest, and their contact with foragers is strictly limited by the particular interaction pattern [67, 68]. Besides, the waste management is one of the primary roles of foragers [69]. Therefore, we consider that the gamergates seldom have access to the nest materials handled by foragers, to which the firmicute symbiont possibly attaches. In parallel with the behavioral aspect, the physiological differentiation within a colony should also be considered for controlling the titer of firmicute symbiont. Previous studies revealed higher transcription levels of several genes involved in nutrition as well as reproduction in the abdomen of gamergate, compared to those of workers [33, 70, 71]. In addition to the behavioral patterns and spatial distributions, such a specific physiological state of gamergate might further reduce the opportunistic infection. Future study should clarify these points, leading to a whole picture of caste-dependent microbiota in this species.

The biological function of the symbiont is also important to explain why the firmicute symbiont is not associated with gamergates from an evolutionary point of view. In spite of the unusual infection pattern of the symbiont, the high prevalence and titer of the firmicute symbiont in workers imply some specific unknown function of the symbiont. Symbiotic bacteria of insects show various biological functions in insect hosts, such as provisioning essential nutrients and preventing antagonists, to enhance the host’s fitness [3, 72]. In ants, *Camponotus* species harbor *Blochmannia* bacteria that supply essential amino acids and recycle nitrogen for hosts [11]. The symbiotic bacterial community of *Cephalotes* ants are also involved in the nitrogen recycling process, which supplies essential amino acids to the herbivorous ant hosts [73]. These nutritional roles of symbiotic bacteria may be potentially important in *D*. cf. *indicum*, although these ants are a predacious species that feed on a largely protein-rich diet. Alternatively, considering the fact that the symbiont is mainly associated with foragers, the biological function of the firmicute symbiont is probably related with their tasks, such as foraging and colony defense. Although highly speculative, it would give an advantage to foragers if the firmicute symbiont can prevent pathogens and/or parasitic fungi that frequently contaminate insect carcasses. Another possibility is that the firmicute symbiont would impose some costs on infected gamergate, such as reduced reproductive fitness. To our knowledge, functional differences of the same bacteria between reproductive and non-reproductive castes has never been reported. However, such a specialization would be a new point of focus to understand the coevolution of the novel ant society-bacterium relationship.

The most remarkable evolutionary innovation of eusocial insects is the reproductive division of labor between the castes, wherein each caste is dedicated to different types of tasks including reproduction, nursing, cleaning, defending, and foraging. Considering diverse biological functions of insect symbionts [3, 4], it might not be too surprising if there are caste (task)-associated symbiotic bacteria that are maintained and transmitted in a caste-dependent manner. In addition, while previous studies have reported the regulation of reproductive division of labor in *D*. cf. *indicum* [32, 71, 74–76], it would be of great interest to investigate a potential role of the firmicute symbiont in the maintenance of social system. Further studies on *D*. cf. *indicum* would contribute to a better understanding of the society-microbe relationship in ants and moreover, of what role microorganisms play in the evolution of eusociality.

## Supplementary information

Supplementary Information
