# Supplementary material for: Worker-dependent gut symbiosis in an ant
Source: ISME Commun. 2021 Oct 28;1:60. doi: 10.1038/s43705-021-00061-9 (PMC9723695; doi:10.1038/s43705-021-00061-9)
Supplement: Supplementary file 1 — Supplementary Information [file 43705_2021_61_MOESM1_ESM.docx]

**Worker-dependent gut symbiosis in an ant**

Hiroyuki Shimoji, Hideomi Itoh, Yu Matsuura, Rio Yamashita, Tomoyuki Hori,

Masaru K Hojo, and Yoshitomo Kikuchi

**Supplementary Information**

*Deep sequencing of 16S rRNA gene*

After whole body of each insect sample was homogenized with a pestle, DNA was extracted from the resulting homogenates using QIAmp DNA mini kit (Qiagen, Hilden, Germany) according to the manufacture’s instruction for “DNA Purification from Tissues” with an additional digestive step before the proteinase treatment; addition of lysozyme (FUJIFILM Wako Pure Chemical Industries, Osaka, Japan) at a final concentration of 2 mg/ml and incubation at 37°C for 1h. The prepared DNA was subjected to PCR amplification of the variable region V4 of bacterial 16S rRNA gene using universal primer pair, 515F with Illumina P5 sequences and 806R with 12-bases indexes and Illumina P7 sequences (Table S3, ref 1), and Q5 Hot Start High-Fidelity DNA polymerase master mix (New England BioLabs, MA, USA), according to the manufacture’s instruction. The PCR conditions were as follows: initial denaturation at 98°C for 90 s, followed by 25 cycles of 98°C for 15 s, 55°C for 30 s, and 72°C for 30 s, and a final extension at 72°C for 2 min. After electrophoresis using 2% agarose gel stained with Novel juice (GeneDirex, NV, USA), the PCR amplicons were excised from the gels and purified using Wizard SV Gel and PCR Clean-Up kit (Promega, WI, USA). The quantity of the purified DNA libraries was measured using Qubit 2.0 Fluorometer (Invitrogen, CA, USA) with Qubit DNA HS assay kits (Invitrogen). Paired-end sequencing for all libraries was performed on Illumina MiSeq sequencer (Illumina) with MiSeq Reagent kit v2 (Illumina) according to the manufacture’s instruction.

Raw sequences data was preprocessed as performed as described previously [2, 3]. After paired-end sequences were assembled using the fastq-join tool in ea-utils version 1.1.2 (https://github.com/ExpressionAnalysis/ea-utils) with default setting parameters, low-quality (<30 Q-score) and chimeric sequences were removed using with the macqiime ver. 1.9.1 and the Mothur program ver. 1.42.3 [4, 5]. The resulting sequences were subjected to taxonomic assignment and clustering based on operational taxonomic unit (OTU) with 3% differences by using RDP classifier ver. 2.11 with a 50% confidence threshold and the macqiime ver. 1.9.1, respectively [6, 4].

*Sanger sequencing of 16S rRNA gene*

All the PCR products (in total 15) were purified with ExoSAP-IT Express PCR Product Cleanup Reagent (Thermo Fisher Scientific) and sequenced using BigDye Terminator version 3.1 Cycle Sequencing Kit (Thermo Fisher Scientific) with each primer, 27F, 926R, and 1492R (Table S3) on a Sanger sequencer, ABI 3130xl DNA Sequencer (Applied Biosystems, CA, USA) according to the manufacturer’s instructions.

*Primer and probe design for qPCR and wFISH*

Specific primers for 16S rRNA gene of the firmicute symbiont were designed based on the sub-cloned and directly sequenced PCR products aligned with both themost similar and rather dissimilar bacterial sequences in the phylum Firmicutes deposited in the GenBank. The alignment was generated by the program MAFFT v7.475 [7]. After manually inspecting gaps and trimming the 5’ and 3’ ends, the sequences were directly used for Neighbor-joining analysis in MEGAX [8] under the maximum composite likelihood nucleotide substitution model and bootstrap test of 1000 replicates, by treating gaps/missing data as partial deletion by the cutoff value of 90%. The tree was used to confirm the coverage of OTU sequences in the phylum, and then selected OTUs were again aligned for finding the firmicute specific primer binding regions manually. The quality and specificity of the primers were further checked by Primer3Plus (https://primer3plus.com/cgi-bin/dev/primer3plus.cgi) and Primer-BLAST (https://www.ncbi.nlm.nih.gov/tools/primer-blast/). The oligonucleotide probes were initially designed likewise by referring to the *in situ* accessibility of *Escherichia coli* and other bacteria [9], but we chose exactly the same binding site used in the previous study [10] with only a single nucleotide mismatch, in addition to adjacent helper oligonucleotides (Table S3).

*Statistical analysis*

First, we compared dissimilarity of community structure of microbiota among castes (forager, gamergate, and male) by PERMANOVA using *adonis* in R package vegan [11]. For this analysis, we chose Bray-Curtis and weighted unifrac distance obtained by the macqiime ver. 1.9.1. [4], carried out pairwise comparisons in each distance, and generated Principal Coordinate Analysis (PCoA) plots. We applied 10,000 permutations to calculate *P* value in PERMANOVA, and we used an adjusted *P* value (α = 0.05/3 = 0.017) by Bonferroni correction to decide statistically significance. Next, we compared copy number of the firmicute symbiont between the worker and the gamergate by linear mixed model (LMM) using *lmer* in R package lme4 [12]. We applied logarithmic-transformed values to the LMM. We set caste as a fixed factor and colony ID as a random effect, and the effects of fixed factor are compared with a null model (Type II ANOVA). Also, we carried out a comparison between the nurses and the foragers using the same method as above. Finally, we calculated effect sizes based on Nakagawa et al. [ref. 13] using *r.squaredGLMM* in R package MuMIn [14], and confirmed that all constructed LMMs had neither over nor under dispersion by *testDispersion* in R package DHARMa [15]. All statistical analyses are carried out by R 4.0.3 [16].


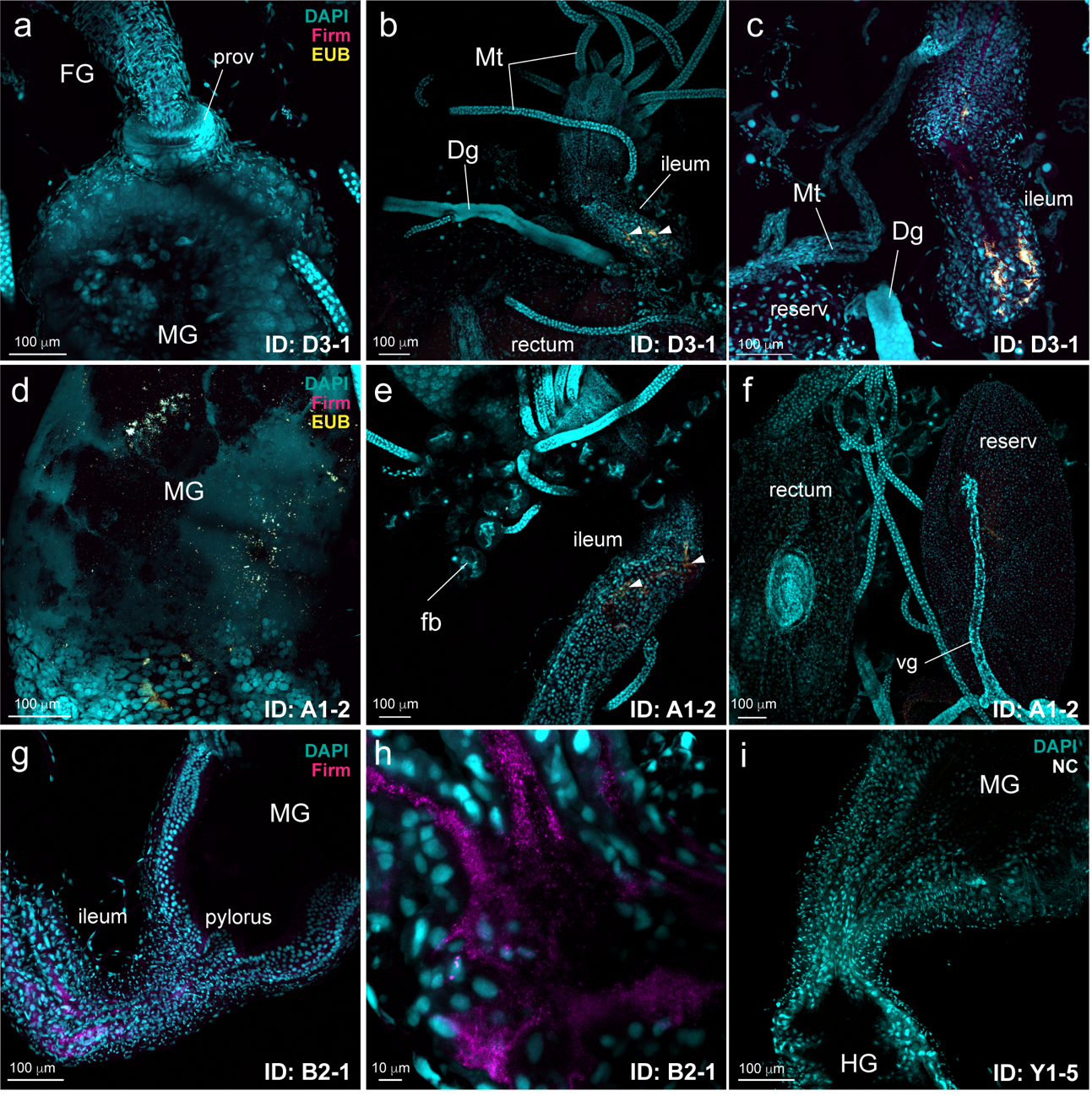


Figure S1. Representatives of wFISH results of the firmicute symbiont using multiple foragers of *D.* cf. *indicum.* Forager individual ID; a-c: D3-1, d-f: A1-2, g and h: B2-1, i: Y1-5.

a; A merged confocal image at the junction between a foregut and midgut, in which no signal was detected neither by the firmicute symbiont-specific probe (Firm), magenta nor the universal eubacteria probe (EUB), yellow. DAPI staining is shown as cyan. b; Another confocal merged image of the same individual detecting both Firm and EUB signals (arrowheads). c; A close-up image of the ileum, Malpighian tubule, venom reservoir and Dufour’s gland. d; An individual showing eubacterial signals in the midgut. e; the same ant harbors gut bacteria in the ileum as overlapping signals of Firm and EUB. f; no signal detected in the rectum and venom reservoir. g; lumen of the midgut containing no firmicute cells, while it can be found near the entrance of the ileum. h; firmicute symbiont cells dispersed in the lumen of the ileum. i; A confocal image of the negative control individual hybridized with positive fluorescently labeled probes and competed with negative unlabeled probes in extremely high (x50) concentration. Abbreviations: Dg, Dufour’s gland; fb, fat body tissues; FG, foregut; HG, hindgut; MG, midgut; Mt, Malpighian tubules; prov, proventriculus; reserv, venom reservoir; vg, venom gland.

**Reference:**

**1.** Caporaso JG, et al. Ultra-high-throughput microbial community analysis on the Illumina HiSeq and MiSeq platforms. ISME J. 2012; 6: 1621–1624.

**2.** Itoh H, et al. Bacterial population succession and adaptation affected by insecticide application and soil spraying history. Front Microbiol. 2014; 5: 457. doi:10.3389/fmicb.2014.00457

**3.** Itoh H, et al. Evidence of environmental and vertical transmission of *Burkholderia* symbionts in the oriental chinch bug *Cavelerius saccharivorus* (Heteroptera: Blissidae). Appl Environ Microbiol. 2014; 80: 5974–5983.

**4.** Caporaso JG, et al. QIIME allows analysis of high-throughput community sequencing data. Nat Methods. 2010; 7: 335–336.

**5.** Schloss PD, et al. Introducing mothur: open-source, platform-independent, community-supported software for describing and comparing microbial communities. Appl Environ Microbiol. 75;23: 7537–7541.

**6.** Wang Q, Garrity GM, Tiedje JM, Cole JR. Naïve bayesian classifier for rapid assignment of rRNA Sequences into the new bacterial taxonomy. Appl. Environ. Microbiol. 2007; 73: 5261–5267.

**7.** Katoh K, Standley DM. MAFFT multiple sequence alignment software version 7: improvements in performance and usability. Mol Biol Evol. 2013; 30: 772–780.

**8.** Stecher G, Tamura K, Kumar S. Molecular Evolutionary Genetics Analysis (MEGA) for macOS. Mol Biol Evol. 2020; 37: 1237–1239.

**9.** Behrens S, Rühland C, Inácio J, Huber H, Fonseca A, Spencer-Martins I *et al*. *In situ* accessibility of small-subunit rRNA of members of the domains Bacteria, Archaea, and Eucarya to Cy3-labeled oligonucleotide probes. Appl Environ Microbiol. 2003; 69: 1748–1758.

**10.** Łukasik P, et al. The structured diversity of specialized gut symbionts of the New World army ants. Mol Ecol. 2017: 26; 3808–3825.

**11.** Oksanen J, et al. vegan: Community Ecology Package. R package version 2.5-7. 2020. https://CRAN.R-project.org/package=vegan

**12.** Bates D, Maechler M, Bolker B, Walker S. Fitting Linear Mixed-Effects Models Using lme4. J Stat Softw. 2015; 67: 1–48.

**13.** Nakagawa S, Johnson PCD, Schielzeth H. The coefficient of determination R2 and intra- class correlation coefficient from generalized linear mixed-effects models revisited and expanded. J R Soc Interface*.* 2007; 14: 20170213.

**14.** Bartoń B. MuMIn: Multi-Model Inference. R package version 1.43.17. 2020.　https://CRAN.R-project.org/package=MuMIn

**15.** Hartig F. DHARMa: Residual Diagnostics for Hierarchical (Multi-Level / Mixed) Regression Models. R package version 0.4.0. 2021. https://CRAN.R-project.org/package=DHARMa

**16.** R Core Team. R: A language and environment for statistical computing. R Foundation for Statistical Computing. 2020; Vienna, Austria. URL https://www.R-project.org/.

**17.** Oh C, et al. Cloning, purification and biochemical characterization of beta agarase from the marine bacterium *Pseudoalteromonas* sp. AG4. J Ind Microbiol Biotechnol. 2010; 37: 483–494.

**18.** Parada AE, Needham DM, Fuhrman JA. Every base matters: assessing small subunit rRNA primers for marine microbiomes with mock communities, time series and global field samples. Environ Microbiol. 2016; 18: 1403–1414.

**19.** Kikuchi Y, Hosokawa T, Fukatsu T. Insect-microbe mutualism without vertical transmission: a stinkbug acquires a beneficial gut symbiont from the environment every generation. Appl Environ Microbiol. 2007; 73: 4308–4316.

**20.** Amann RI, Krumholz L, Stahl DA. Fluorescent-oligonucleotide probing of whole cells for determinative, phylogenetic, and environmental studies in microbiology. J Bacteriol. 1990; 172: 762–770.
